# Pacemaker implantation in a COVID‐19 patient. Balancing the patient’s needs and the team’s risk of exposure

**DOI:** 10.1002/joa3.12480

**Published:** 2020-12-14

**Authors:** Barbara Ignatiuk, Fabio Baratto, Jacopo Monticelli, Francesco Bacchion, Giuseppe Maria Marchese, Giampaolo Pasquetto

**Affiliations:** ^1^ Department of Cardiology Ospedali Riuniti Padova Sud Monselice Italy; ^2^ Department of Anesthesia and Intensive Care Ospedali Riuniti Padova Sud Monselice Italy; ^3^ Department of Infectious Diseases Ospedali Riuniti Padova Sud Monselice Italy

**Keywords:** aortic stenosis, atrioventricular block, cardiovascular complications, COVID‐19, pacemaker

## Abstract

COVID‐19 patients may have cardiovascular complications requiring invasive treatment. Pacemaker implantation procedure may be challenging because of the necessity of personal protective equipment use. We report pacemaker implantation in a 78‐year‐old man with severe bilateral COVID‐19 interstitial pneumonia, a second degree 2:1 atrioventricular block, and concomitant aortic stenosis.

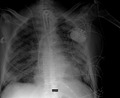

## 1 CASE

**FIGURE 1:**
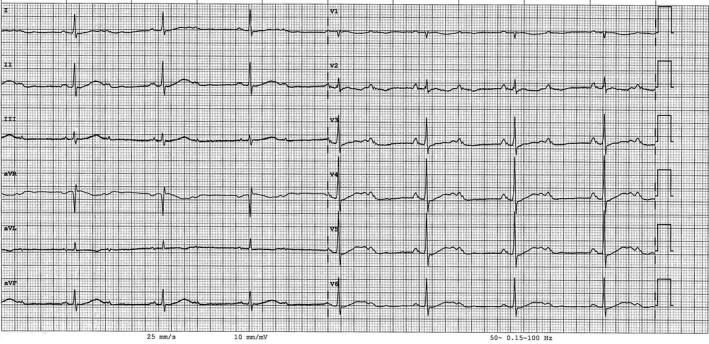
ECG at admission. Sinus rhythm, second degree 2:1 atrioventricular block and ventricular rate of 46 bpm.

**FIGURE 2:**
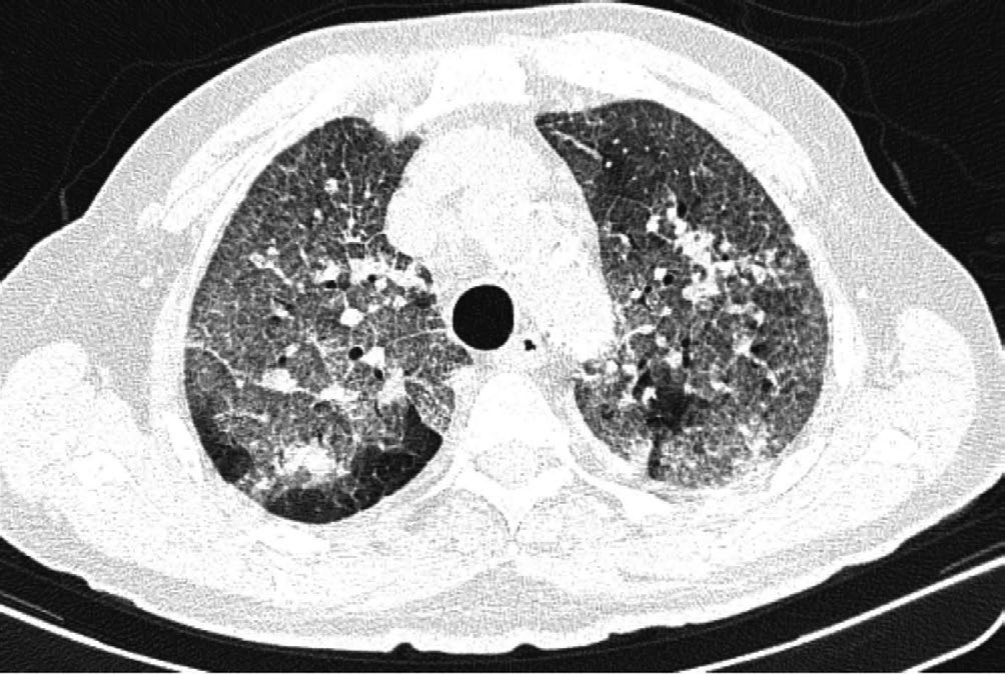
The baseline noncontrast‐enhanced computerized tomography showed wide bilateral parenchymal consolidation areas with ground‐glass opacification, “crazy paving” patterns, and small pulmonary nodules.

A 78‐year‐old man was admitted because of respiratory failure in March 2020, during the COVID‐19 outbreak. He suffered from diabetes, hypertension, dyslipidemia, and moderate aortic stenosis. He had been reporting a weakness for 3 days and worsening dyspnea. In the emergency room, his blood pressure was 93/60 mmHg, heart rate 40 bpm, respiratory rate 28/min, and temperature 35°C. ECG documented a sinus rhythm, second degree 2:1 atrioventricular (AV) block, and ventricular rate of 46 bpm (Figure 1). Arterial blood gas showed acidosis and severe hypoxemia (pH 7.31, pO_2_ 39 mmHg, O_2_ saturation 75% on 8 L/min oxygen). The high‐resolution computed tomography (HRCT) scan revealed a high probability of severe COVID‐19 (Figure 2). A subsequent nasopharyngeal swab for SARS‐CoV‐2 resulted positive. Laboratory analysis showed an important leukocytosis (23.58 × 109/L with 22.49 × 109/L neutrophils), elevated C‐reactive protein (342 mg/dL), and procalcitonin (16.4 μg/L). The patient was intubated, achieving an oxygen saturation of 98.6%. He was stabilized with crystalloids and IV noradrenaline. After blood cultures were performed, he was started on an experimental treatment with azithromycin 500 mg every 24 hours for 5 days and hydroxychloroquine 200 mg TID for 10 days. He was empirically treated with piperacillin/tazobactam. The 2:1 AV block persisted with a heart rate around 45 bpm, and a transient complete AV block (heart rate of 49 bpm). The blood pressure values were stable on isoprenaline. He received one single dose of intravenous tocilizumab 800 mg. All blood cultures resulted negative. The echocardiogram showed nondilated hypertrophic left ventricle with normal ejection fraction, and aortic valve area of 0.6 cm^2^/m^2^.

**FIGURE 3:**
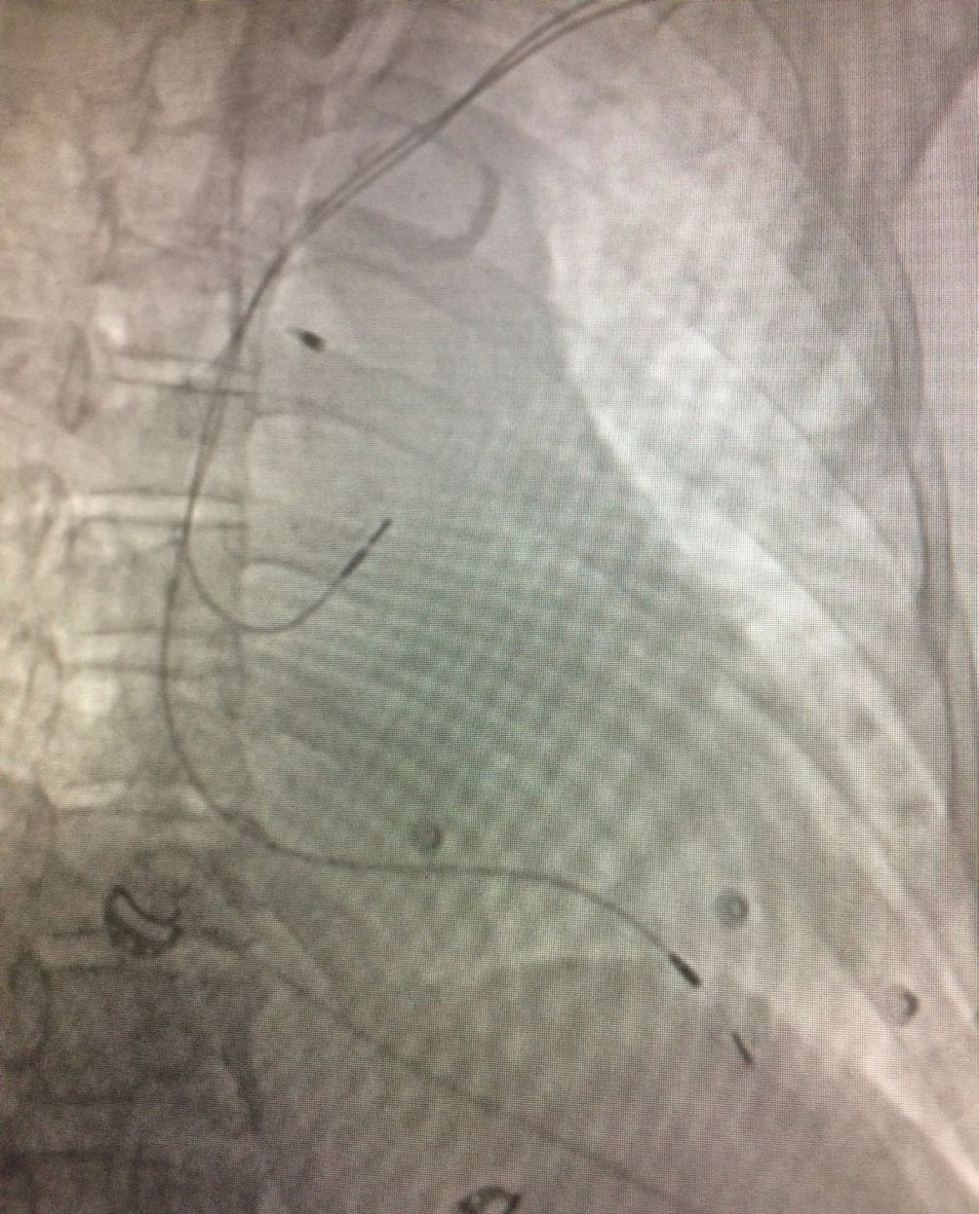
Pacemaker implantation (fluoroscopy image, RAO view). Final leads position: ventricular lead in the right ventricle apex, atrial lead in the right atrial appendage.

**FIGURE 4:**
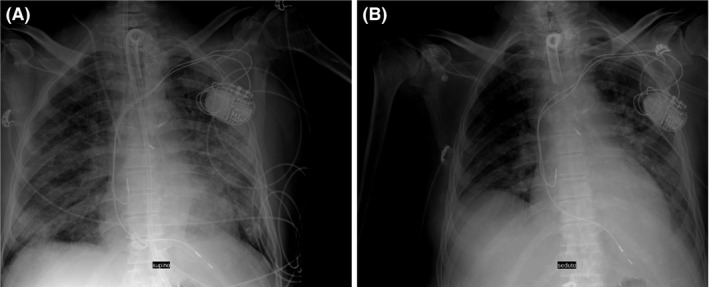
The 1 month (A) and 3 months (B) follow‐up chest X‐ray revealed a progressive recovery from interstitial pneumonia.

Three weeks after admission, when the clinical picture substantially improved, a permanent pacemaker was positioned. The procedure was performed by an experienced operator, two nurses, and an anesthesiologist and was carried out in total intravenous general anesthesia. No other personnel was allowed to access the area. All unnecessary equipment was removed from the operating room. All professionals wore protective suits, FFP3 mask, external surgical mask, eyewear, double gloves, and lead gowns. The implanting physician wore a sterile coat and a double pair of sterile gloves. A dual‐chamber Medtronic pacemaker was implanted without complications. Ventricular and atrial passive leads (Figure 3) were positioned by double left subclavian puncture with optimal pacing and sensing parameters. Antimicrobial envelope (Medtronic Tyrx) was positioned in the pocket. The hemodynamics upon the procedure was stable, as were the biochemical markers. Procalcitonin, C‐reactive protein, and troponin I were normalized. The subsequent 1 and 3 months follow‐up chest X‐ray examinations revealed a progressive recovery from interstitial pneumonia (Figure 4).

The professionals involved in the procedure were swabbed for COVID‐19 every 10 days according to institutional protocols, with negative results over 20 days.

The patient cleared SARS‐CoV‐2 from multiple consecutive respiratory samples and recovered from COVID‐19, but has had several subsequent pneumonias caused by multidrug‐resistant organisms (*Pseudomonas aeruginosa* and *Acinetobacter baumannii*) and was tracheostomized. After 4 months, the patient was still hospitalized to continue physical therapy. Pacing parameters were optimal.

## 2 DISCUSSION

We report a successful pacemaker implantation procedure in a diabetic 78‐year‐old man with respiratory distress caused by severe COVID‐19, concomitant aortic stenosis, and a second degree 2:1 AV block. This case highlights the significance of concomitant pathologies in patients affected by COVID‐19, whose treatment should take into account both the clinical status of the patient and the need for the team's protection from unnecessary exposure.

The AV block is a common complication of aortic stenosis as a degenerative process may easily affect the conduction tissue. An acquired third‐ or second‐degree type 2 block is in class I for pacemaker implantation. In our case, the watchful waiting strategy was adopted. The determinant factor of the clinical state was not a heart rate but a SARS‐CoV‐2 infection with probable concomitant bacterial sepsis. After the initial phase, blood pressure was satisfactory. At the same time, the age and comorbidities placed the patient at increased risk of lethality. The procedure was performed when a favorable evolution was observed and a discharge from the ICU could be hypothesized. Temporary pacing was not necessary because the hemodynamics remained stable. The presence of a femoral pacemaker in a COVID‐19 patient can be problematic because such patients often require ventilation in a prone position, which increases the risk of electrode displacement and perforation.

To the best of our knowledge, this is the first case of transvenous pacemaker implantation reported in literature in a COVID‐19 patient. COVID‐19 patients may develop hypokinetic or hyperkinetic arrhythmias resulting from side effects of drugs, the inflammatory process, or hypoxemia (the potential mechanisms are complex). Two isolated cases of transient AV block are described in the absence of preexisting cardiac disease. One patient died (troponin is not reported), the second one recovered after a short period of temporary pacing. The second patient had an acute myocardial injury with the troponin I >90 000 ng/L and a paroxysmal AV block in the acute phase. Our patient had associated aortic stenosis and AV block is common in that condition. It is possible that concomitant COVID‐19 might have additionally compromised the conduction tissue. Troponin levels were only slightly elevated: 195 on admission, 9 before implantation. The AV block persisted until the procedure. In our opinion, it was mainly an evolution of the aortic stenosis, maybe exacerbated by COVID‐19 but without frank myocarditis.

To operate on an intubated patient was judged safer for the staff (avoided aerosol formation) and for the patient (pneumothorax possibility during the subclavian puncture).

Worldwide experience has demonstrated the high transmission potential of this pathogen in the health‐care setting. Several professional boards provided general rules for the management of cardiovascular disease during the COVID‐19 pandemic to minimize contamination risks. Surgical procedures on COVID‐19 patients should be postponed whenever possible until confirmed infection clearance.

Pacemaker implantation normally is not a very complicated procedure. The difficulty resulted from the particular context, operating with full personal protective equipment. Attention must be paid to protect the team and avoid compromising sterility. Any invasive strategy in COVID‐19 patients should be carefully evaluated in terms of indication, timing, and perioperative management.

## CONFLICTS OF INTEREST

None declared.

